# The Study of Field Equivalence Determined by the Modeled Percentage Depth Dose in Electron Beam Radiation Therapy

**DOI:** 10.1155/2021/3397350

**Published:** 2021-10-08

**Authors:** You-Guo Ma, Yan-Shan Zhang, Yan-Cheng Ye, Jia-Ming Wu

**Affiliations:** ^1^Heavy Ion Center of Wuwei Cancer Hospital, Gansu Wuwei Academy of Medical Sciences, Gansu Wuwei Tumor Hospital, Wuwei City Gansu Province, China; ^2^Department of Medical Physics, Chengde Medical University, Chengde City, Hebei Province, China; ^3^Department of Radiation Oncology, Yee Zen General Hospital, Taoyuan City, Taiwan

## Abstract

**Introduction:**

This study presents an empirical method to model the curve of electron beam percent depth dose (PDD) by using the primary-tail function in electron beam radiation therapy. The modeling parameters *N* and *n* can be used to predict the minimal side length when the field size is reduced below that required for lateral scatter equilibrium (LSE) in electron radiation therapy.

**Methods and Materials:**

The electrons' PDD curves were modeled by the primary-tail function in this study. The primary function included the exponential function and the main parameters of *N* and *μ*, while the tail function was composed of a sigmoid function with the main parameter of *n*. The PDD of five electron energies was modeled by the primary and tail function by adjusting the parameters of *N*, *μ*, and *n*. The *R*_50_ and *R*_p_ can be derived from the modeled straight line of 80% to 20% region of PDD. The same electron energy with different cone sizes was also modeled by the primary-tail function. The stopping power of different electron energies in different depths can also be derived from the parameters *N*, *μ*, and *n*.

**Results:**

The main parameters *N* and *n* increase but *μ* decreases in the primary-tail function for characterizing the electron beam PDD when the electron energy increased. The relationship of parameter *n*, *N*, and ln(−*μ*) with electron energy are *n* = 31.667*E*_0_ − 88, *N* = 0.9975*E*_0_ − 2.8535, and ln(−*μ*) = −0.1355*E*_0_ − 6.0986, respectively. Percent depth dose was derived from the percent reading curve by multiplying the stopping power relevant to the depth in water at a certain electron energy. The stopping power of different electron energies can be derived from *n* and *N* with the following equation: stopping power = (−0.042ln(*N*_*E*_0__) + 1.072)*e*^(−n_E_0__ · 5 · 10^−5^ + 0.0381)·*x*^, where *x* is the depth in water. The lateral scatter equivalence (LSE) of the clinical electron beam can be described by the parameters *E*_0_, *n*, and *N* in the equation of S_eq_ = (*n*_*E*_0__ − *N*_*E*_0__)^0.288^/(*E*_0_/*n*_*E*_0__)^0.0195^. The LSE was compared with the root mean square scatter angular distribution method and shows the agreement of depth dose distributions within ±2%.

**Conclusions:**

The PDD of the electron beam at different energies and cone sizes can be modeled with an empirical model to deal with what is the minimal field size without changing the percent depth dose when approximate LSE is given in centimeters of water.

## 1. Introduction

The electron beam has the advantage in the target volume of superficial tumors such as skin cancers, breast cancer for chest wall irradiation, node boost dose, and head and neck cancers, while the minimizing dose to steeper tissues is widely adopted in clinical use.

The shape of the depth dose curve characteristic is the major attraction of the electron beam which offers a distinct clinical advantage over the conventional X-ray modalities for the superficial lesion. The depth dose curves, beam profiles, absolute output, and cone factor with different electron energy and cone sizes must be measured and implemented to the treatment planning system before the electron beam can be applied for clinical use. The electron dose distribution calculation algorithms mainly use the pencil beam model [1, 2] or another numerical model [3, 4] in commercial treatment planning systems [5]. The parameters of the model in pencil beams or Monte Carlo treatment planning system for electron beams need to be adjusted to fit the measurement of clinical data to perform accurate dosimetry calculation and prediction of PDD.

The pencil beams or Monte Carlo simulation algorithm describes PDD by using a complex theory with special function and *σ*_r_(*z*) to calculate dose at any depth of *z* [6].

Some investigators used the pencil beams or Monte Carlo simulation algorithm to investigate the changes of PDD affected by the reduction of field size smaller than the lateral scatter equilibrium (LSE) [7]. But the output factor does not change coincidently with field size increased since the PDD initially increases but finally comes to constant when the LSE is reached.

The motivation of this study we are concerned with was to reexamine the problem proposed skeptically by some investigators [8] that it was hard to find a central axis depth dose distribution just the same as any given arbitrary field in the electron beam. Fortunately, the other investigators [9] provided a solution to find the same PDD for a given electron field.

They empirically used electron energy to define the LSE, which means the depth dose distribution becomes independent of field size; therefore, we would like to visit the problem and try to find a simple way to define the minimum side length, S_eq_, for the establishment of LSE at all depths merely by using the parameters used by the mathematic model in this study.

In this study, we proposed a simple mathematic equation to model the PDD by using the primary-tail function. Besides, we also used this empirical model to investigate the effects of field size on the central axis depth dose curve when the distance between the point of measurement and the edge of the field is shorter than the range of the laterally scattered electrons.

## 2. Materials and Methods

### 2.1. Electron Percent Depth Dose Numerical Equation

The primary-tail model originated from the proportion function *y*(*x*) = 1/*x*. When *x* increases from -∞ to 0, the curve of *y* is located in the region of the (-,-) quadrant. When *x* goes from 0 to +∞, the curve of *y* is located in the region of the (+, +) quadrant. Let 1/*x* be 1/∣*x*∣, then the curve falls in the (-,+) and the (+,+) quadrants. The curve of *y*(*x*) = 1/∣*x*∣ has a left and a right tail of the dose-profile-shape pattern. Let $y\left(x\right)=1/|x|=1/\sqrt{\left(x^{2}\right)}$. When *x* = 0, *y* becomes infinite which does not happen in real dose profiles. Therefore, we insert *n* into *y*(*x*) to be $\text{tail}\left(x\right)=\left(1/\sqrt{n+\left(x^{2}\right)}\right)$, where *n* > 0, let $\text{tail}\left(x\right)=\left[\left(1-x/\sqrt{n^{0.5}+x^{2}}\right)+t\right]$, where *x* is the depth in water in the unit of mm, *n* is a spreading factor of real number scalar, *t* is a real number scalar to fine tune the height of the X-ray contamination.

On the other hand, the function *f*(*x*) = (*x*^0.1^/(*N* + *x*^0.2^)) demonstrates ascending value *f* with an increasing depth of *x* in water. When an exponential function *e*^*μx*^ to *f*(*x*) is introduced, the combination becomes the primary function (*x*^0.1^/(*N* + *x*^0.2^))*e*^−*μx*^, namely, primary(*x*) = (*x*^0.1^/(*N* + *x*^0.2^))*e*^−*μx*^, where *x* is the depth in water in the unit of mm and *N* > 0 and is a harden factor of real number scalar. When *x* = 0, *N* plays an important role to avoid primary(*x*) from becoming infinite, while *μ* is the linear attenuation factor for fine tuning the growth of the (*x*^0.1^/(*N* + *x*^0.2^)) value.

Finally, the primary-tail model can be expressed as follows:

(1) $$\begin{array}{c} {\text{PDD}}_{p-t}=\left(\frac{x^{0.1}}{N+x^{0.2}}\right)e^{-\mu x}\cdot \left[\left(1-\frac{x}{\sqrt{n^{0.5}+x^{2}}}\right)+\text{t}\right]. \end{array}$$

There are two numerical equations for describing the percent depth dose curves: the primary function and the tail function. The primary function is described as follows:

(2) $$\begin{array}{c} \text{Primary function}:\left(\frac{x^{0.1}}{N+x^{0.2}}\right)e^{-\mu x}, \end{array}$$

where *x* is a real number on the horizontal axis in the unit of mm and also denotes as the depth in water in the unit of mm, *N* is a scalar of harden factor, and *μ* is the linear attenuation factor in the unit of mm^−1^. The only scalar of the parameters *x*, *N*, and *μ* are replaced for calculation in the primary function.

(3) $$\begin{array}{c} \text{Tail function}:\left[\left(1-\frac{x}{\sqrt{n^{0.5}+x^{2}}}\right)+t\right], \end{array}$$

where *x* is the same definition in equation (1) while *n* is a spreading factor and *t* is a factor for adjusting the height of the tail. The only scalars of the parameters *x*, *n*, and *t* are replaced for calculation in the tail function.

The empirical function of percentage depth dose is the combination of these two functions, denoted as PDD_*p*−*t*_:

(4) $$\begin{array}{c} {\text{PDD}}_{p-t}=\left(\frac{x^{0.1}}{N+x^{0.2}}\right)e^{-\mu x}\cdot \left[\left(1-\frac{x}{\sqrt{n^{0.5}+x^{2}}}\right)+t\right]. \end{array}$$

All percentage depth doses of five electron energies with different cone sizes at SSD = 100 cm were adjusted by the main parameters of *N*, *n*, and *μ* to get the best fitting.

### 2.2. Experiment Design and Steps

The experiment was conducted in the following steps by using the electron beam provided by our institute's linear accelerator Varian VitalBeam (Varian, Palo Alto, CA, USA) and Elekta Infinity (Elekta, Stockholm, Sweden):

1. The measurement of electron beam ionization depth dose curves of five energies at SSD = 100 cm with different cone sizes and different electron cutouts was conducted by parallel plate chamber (PTW Freiburg, Germany, TM 23343-3765) in a 3-dimensional water phantom
2. The measurement of electron beam percent depth dose curves of five energies at SSD = 100 cm with different cone sizes and different electrons was conducted by using the Gafchromic EBT3 films (Ashland Specialty Ingredients GP, NJ, USA; Lot # 04022001) in a solid water phantom
3. The empirical modeling of the electron beam percent depth dose curves was characterized by the primary-tail function
4. Logistic regression of the empirical modeling parameters of *N*, *μ*, and *n* was made for the best fitting in the primary-tail function
5. The final step was to find a simple way to define the minimum side length, S_eq_, to establish the LSE at all depths merely by using the parameters *N*, *n*, and *E*_0_ used by the mathematic primary-tail model in this study

The details of each step are described in the following sections.

### 2.3. The Comparison of Depth Dose Curve Converted via the ionization curves measured by Farmer chamber with Gafchromic EBT3 Film

We used Gafchromic EBT3 films for the depth dose curve measurement for determining the percent depth dose measurement. The film measurements followed international protocols. A preexposure technique was used for the derivation of the calibration curve [10–12]. This was performed by giving each film a priming dose of 2 Gy to homogenize the film density using the facility of Wuwei Heavy Ion Center, Cancer Hospital (WHICH), Gansu, China, with a dose of 1 Gy at the electron energy of 12 MeV. We then measured the dose homogeneity using a densitometer. Graded doses of 10 cGy with an interval of 20 cGy to 200 cGy were given to the GAF chromic film to obtain the Hurter-Driffield calibration curve (H-D curve). The film was sandwiched by the solid water phantom and was irradiated with the film surface parallel to the beam central axis at SSD 100 cm for different cone sizes. The substance of Gafchromic provided by the vendor was assumed to be water equivalent.

All exposed films of the depth dose curve were then scanned with an Epson Expression 11000XL scanner, and the data were saved as tagged image file format (TIFF) and analyzed by the VeriSoft imaging procession software. A red filter was placed on top of the GAF films to increase the resolution of the dose-OD curves [13].

The depth dose curve derived from the ionization depth curve from the parallel-plate chamber was then compared with the depth dose curves measured by Gafchromic EBT3 films.

Absolute output and machine quality assurance were performed before conducting the measurements of percent ionization depth by parallel-plate chamber, and the percent depth dose curve was measured by the Gafchromic EBT3 film.

### 2.4. Ionization Depth Curve Measurement

A total of five electron energies from 6 MeV to 18 MeV with an increasing interval of 3 MeV of Varian VitalBeam linear accelerator at SSD = 100 cm and cone sizes varying from 6 cm × 6 cm, 10 cm × 10 cm, 15 cm × 15 cm, 20 cm × 20 cm to 25 cm × 25 cm for the measurements of depth ionization curves were carried out at WHICH in this study. Since the parallel-plate chamber has a small plate separation and the charged electron particle fluence is mostly forward-directed, it is explicit that the point of measurement is the front surface of the cavity. The type of parallel-plate chamber used in this study was PTW TM 23343-3765, and the effective point of measurement was 0.3 mm upstream shift according to the vendor's suggestion. PTW 3D water phantom (PTW Freiburg, T41029-00006) was used for the percent ionization depth curve measurements. The depth curve measured by PTW TM 23343-3765 parallel-plate chamber was then derived to percent depth dose with the conversion function provided by the water phantom, and this percent depth dose can then be compared with the percent depth dose curve measured by the film.

For the electron, the incident of the monoenergetic spectrum is degraded as it penetrates the water, the restricted stopping power, (*L*/*ρ*)_air_^med^, increases significantly with depth. In this study, the stopping power can be derived by the *N* and *n* relative to its electron energy in the following equation:

(5) $$\begin{array}{c} \text{Stopping power}=\left(-0.042\ln\left(N_{E_{0}}\right)+1.072\right)e^{\left(-n_{E_{0}}\cdot 5\cdot 10^{-5}+0.0381\right)\cdot x}, \end{array}$$

where *N*_*E*_0__ and *n*_*E*_0__ are the *N* and *n* at the electron energy *E*_0_, while *x* is the depth in water in the unit of mm.

The relationship between *μ* and the slope can be calculated by the following equation:

(6) $$\begin{array}{c} \tan^{-1}\left(\text{slope}\right)=-6.6729\cdot \ln\left(\mu \right)-16.623. \end{array}$$

The slope decrease when *μ* increases, which means the larger the electron energy, the less the side scatter is.

According to the previous investigator's study, the minimum side length for square fields, S_eq_, to establish the LSE at all depths, is given by the equations of *S*_*eq*_:

$S_{eq}=1.58\cdot \sqrt{E_{0}}.$

In our study, the minimum side length without changing the standard percent depth dose is given by the equation S_eq_:

(7) $$\begin{array}{c} {\text{S}}_{\text{eq}}=\frac{{\left(n_{E_{0}}-N_{E_{0}}\right)}^{0.288}}{{\left(E_{0}/n_{E_{0}}\right)}^{0.0195}}. \end{array}$$

For the specification of most probable energy,(*E*)_0_is defined by the Nordic Association of Clinical Physics [14] as the position of the electron fluence*ϕ*versus the energy spectral peak at the phantom surface [14] and the use of the following relationship listed:(Please romove the [14] of Physics [14] and place behind phantom surface)

(*E*)_0_ = *C*_1_ + *C*_2_*R*_p_ + *C*_3_*R*_p_^2^, where *R*_p_ is the practical range in centimeters. For water, *C*_1_ = 0.22 MeV, C_2_ = 1.98 MeV/cm, and C_3_ = 0.0025 MeV/cm^2^.

The practical range, *R*_p_, is the depth of the point where the tangent to the descending linear portion of the curve (at the point of inflection) intersects the extrapolated background.

The deviation of modeling *PDD*_*P*−*t*_ with measured PDD at a certain depth was defined as

(8) $$\begin{array}{c} \left(\frac{\text{the dose of modeling }{\text{PDD}}_{P-t}\text{ at a certain depth}-\text{the dose of measured PDD at the same depth}}{\text{the dose of measured PDD at the same depth}}\right)\times 100\%. \end{array}$$

## 3. Results

A good agreement of PDD measured by film and the PDD measured by water phantom converted by the parallel-plate ion chamber has been observed in this study. It is proven that the energy independence of the film which may be due to the collision stopping power in emulsion and in water varies slowly with the electron energy.

### 3.1. The Best Fitting of Percent Depth Dose by Empirical Function in Five Electron Energies

**Figure 1:**
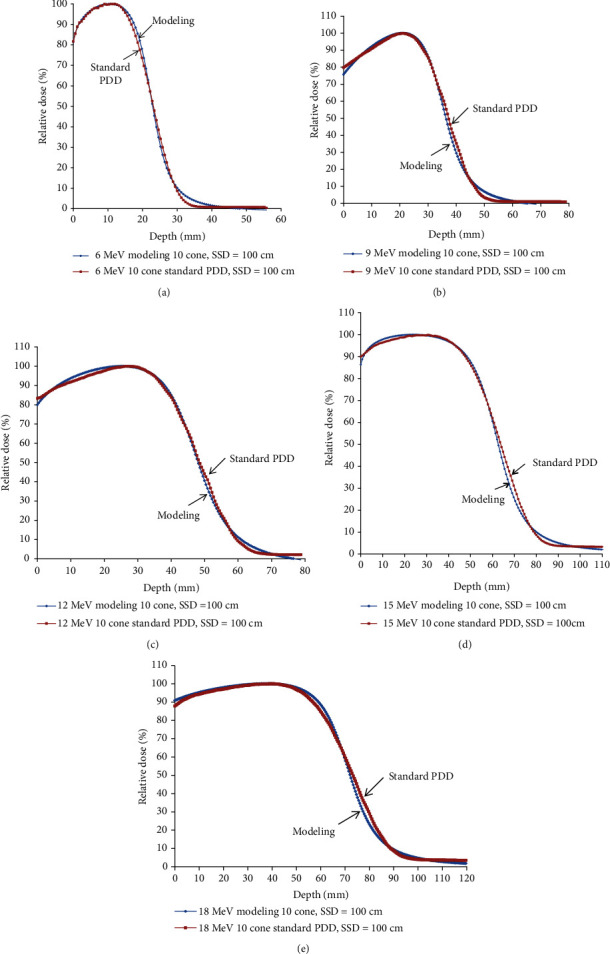
(a–e) Represents the fitting of the percent depth dose curve by adjusting the main parameters of *N*, *n*, and *μ* in the electron energy from 6 to 18 MeV, representatively.

The percent depth dose of the electron beam with different energies adopted in this study was already measured by the water phantom at the commission of the linear accelerator. By adjusting the main parameters of *N*, *n*, and *μ*, we get the best fitting of all-electron percent depth dose curves of every energy with cone size 10 cm × 10 cm in Figure 1.

### 3.2. The Percent Depth Dose Was Fitted by Empirical Function in the Same Electron Energy with Different Cone Sizes

The percent depth dose varies slightly in the same electron energy with different cone sizes; therefore, we tabulated the deviation of measured and modeled with one of the electron energy of 12 MeV.

Table 1(a) demonstrates the comparison in fitting the same electron energy of 12 MeV in 5 different cone sizes from 6cm × 6 cm to 25 cm × 25 cm at SSD = 100 cm.

The comparison error between the modeling and the measurements for different electron energies but the same electron size of 10 cm × 10 cm is listed in Table 1(b).

### 3.3. The Best Fitting of Percent Depth Dose by the Main Parameters of *N*, *n*, and *μ* in the Empirical Function of All-Electron Energy with Different Cone Sizes

Table 2 shows the main parameters of *N*, *n*, and *μ* of the best fitting of all-electron percent depth dose curves at every energy with different cone sizes. According to Table 2, *N* and *n* increases while *μ* decreases when electron energy increases.

By using the primary-tail function for modeling electron energy at 12 MeV in different cone sizes, the measured PDD can be characterized perfectly than the other electron energies. For the overall fitting of the measured PDD, we found that the intersection of the X-ray contamination with the PDD descending portion at the turning point was pretty bad which was the limitation of the primary-tail function. It might be caused by a lack of scattering algorithm consideration at the junction of the X-ray contamination and the end of the PDD curve in the current PDD_*P*−*t*_ model. The solution to this problem will be the next study topic in our future investigation.

## 4. Discussion

The percent depth dose can be fitted quite well with the primary-tail modeling by adjusting the main parameters of *N*, *n*, and *μ* in five electron energies except for the descending curve intersection area with X-ray contamination in Figure 1.

Tables 1(a) and 1(b) demonstrate the comparison in fitting the electron energy of 12 MeV with 5 cone sizes from 6cm × 6 cm to 25 cm × 25 cm at SSD = 100 cm while the ranges of error between the modeling and the measurements for different electron energies at the same cone size of 10 cm × 10 cm are listed in Table 1(b). A significant error between the model and the measurements was found at the turning point of the PDD curve's descending portion intersecting with the X-ray contamination while this is irrelevant when examined the *R*_p_, but it is a limitation of the primary-tail model indeed.

The impact in clinical of the large deviation at the turning point of the PDD descending potion to X-ray contamination intersection is insignificant when examining *R*_p_. Secondly, the purpose of this study is to investigate the field equivalence in the electron beam; the disagreement at the turning point is located at the end of the central axis depth dose curve; therefore, the influence of the large deviation to the point of measurement and the shorter edge than the range of the laterally scattered is irrelevant. Finally, the monitor unit calculation is indifferent to the presence of the large deviation at the turning point since 90% isodose curve is usually selected for MU calculation for dose delivery in electron beam therapy.

The more electron energy, the more *N*, *n*, and *μ* it has for the best fitting of the PDD curve as shown in Table 2.

**Figure 2:**
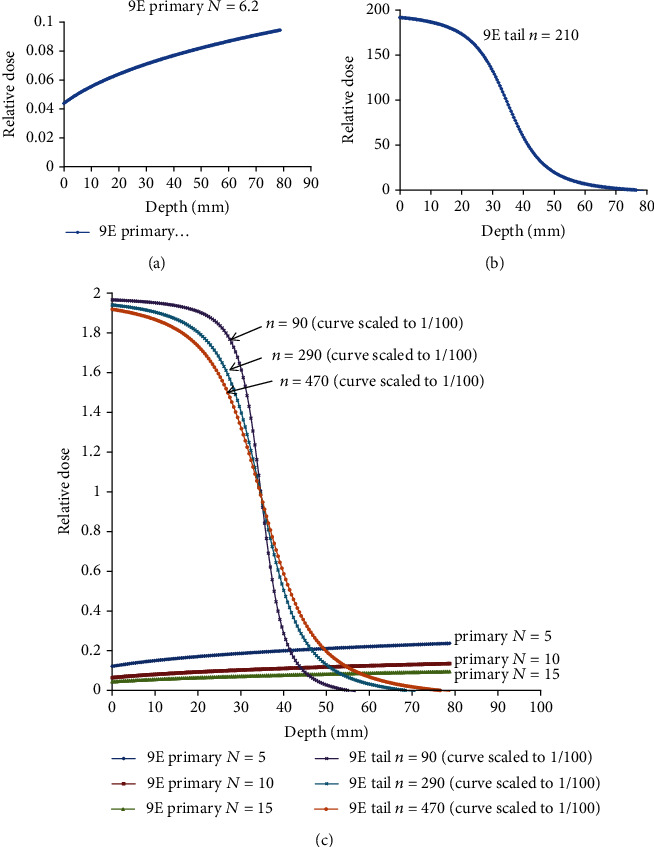
Demonstrates how the primary function is affected by parameters *N* and *n*; the best fitting of parameter *N* and *n* in the PDD of 9 MeV electron beam in (a) and (b). The curve becomes less attenuated in a large *N* as shown at the bottom in (c). The factor *n* represents the spreading factor at any electron energy; it is explicitly a large electron beam that comes with a large *n* as shown in Table 2 and (c). The factor *μ* is used to fine-tune the shape of the PDD curve, and it is trending to be smaller in a high electron energy beam.

Figures 2(a) and 2(b) show the best fitting of parameters *N* and *n* in the PDD of 9 MeV electron beam, respectively. We can adjust the value of *N* to fit the measured PDD. Since *N* is the hardened factor, more electron energy comes with a large value of *N* as shown in Table 2, and the curve becomes less attenuated in a large *N* as shown at the bottom in Figure 2(c). The factor *n* represents the spreading factor at any electron energy; it is explicitly a large electron beam that comes with a large *n* as shown in Table 2 and Figure 2(c). The factor *μ* is used for fine-tuning the shape of the PDD curve, and it is trending to be smaller in a high electron energy beam as shown in Table 2.

In this study, we defined the practical range, *R*_p_, as the depth of the point where the tangent to the descending linear portion of 80% to 20% of the curve intersects the extrapolated X-ray contamination at the point of inflection. The depth at which the dose is 50% of the maximum dose is defined as *R*_50_, and the mean energy of the electron beam, *E*_0_, is equaled to *R*_50_ × 2.33 MeV/cm.  Table 3 shows the maximum reading depth, dose maximum depth, and mean energy of the electron beam, *E*_0_ (*E*_0_ = 2.33 MeV/cm · *R*_50_). The depth at which the dose is 50% and 90% of the maximum dose is defined as *R*_50_ and *R*_90_, respectively, as the American Association of Physicists in Medicine (AAPM) protocol recommended [15] in this study.

**Figure 3:**
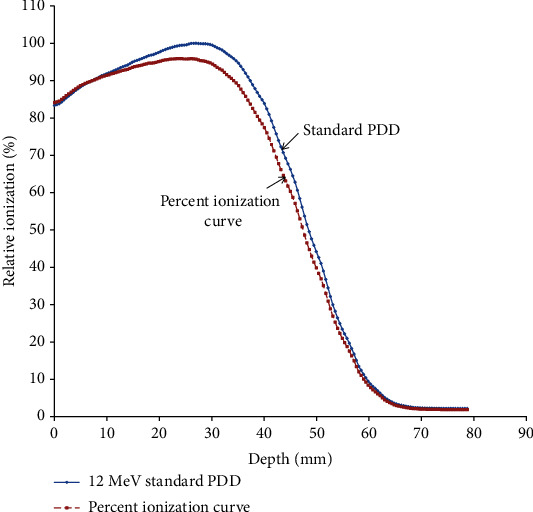
The converted ionization depth curve was calculated by dividing the water phantom of PDD at 10 cones with the relative stopping power from the above calculation at any certain depth for 12 MeV.

The depth dose curve converted by ionization depth curve was calculated by dividing the standard PDD measured by water phantom with the relative stopping power at a certain depth for 12 MeV and is shown in Figure 3.

The angle of the descending portion ranged in between 80% and 20% of cone size 10 cm × 10 cm at any electron energy is defined as the slope of the electron PDD.

**Figure 4:**
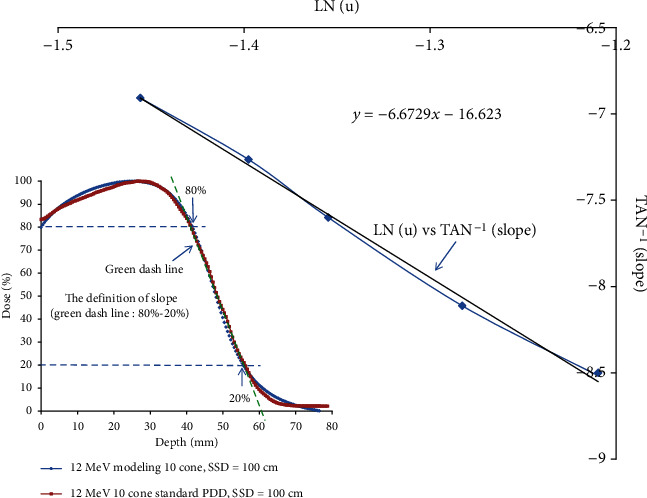
The relationship of the slope of descending portion ranged in between 80% and 20% of the electron PDD at cone size 10 cm × 10 cm is relative to the electron energy. The relationship between *μ* and slope can be calculated by the equation, tan^−1^(slope) = −6.6729 · ln(−*μ*) − 16.623; as the figure shows, the slope decreases when the *μ* increases, which means the larger the electron energy, the less the side scatter it is.

The relationship between *μ* and slope is shown in Figure 4 and can be calculated by equation ((5). The modeled percent depth dose curve as well as the standard percent depth dose curves varied by a little deviation with the field size at the same electron energy of 12 MeV in Table 1(a).

In electron beam therapy, if the X-ray jaw setting were changed with the treatment field, the percent depth dose curve would vary a wide range with field size, especially for lower energy beams. In clinical practice, electron therapy usually provides a fixed jaw opening, and the treatment field size is varied by various cone sizes. Such an arrangement minimizes the variation of collimator scatter, and therefore, the PDD variation with field size is kept reasonably small if the field size is not reduced below that required for lateral scatter equilibrium (LSE).

The effects of field size on output and the change of PDD due to phantom scatter is significant when the field is shorter than the range of the laterally scattered electrons. After this distance is reached, there is no further increase in depth dose caused by phantom scatter. When the field size of electron cutout is reduced below that required for LSE, the change of PDD is obvious and the dose rate decreases rapidly as well. The change of PDD is shown in Figure 4. In these measurements, the field size at the phantom surface was fabricated by electron cutout, which, therefore, varied without changing the photon jaw setting. For small fields, the shape of the depth dose, as well as output factor, can be significantly reduced compared with the broad beam distribution.

Figure 4 shows that the relationship between *μ* and the slope can be calculated by equation (5); the slope decreases when the *μ* increases, which means the larger the electron energy, the less the side scatter is.

**Figure 5:**
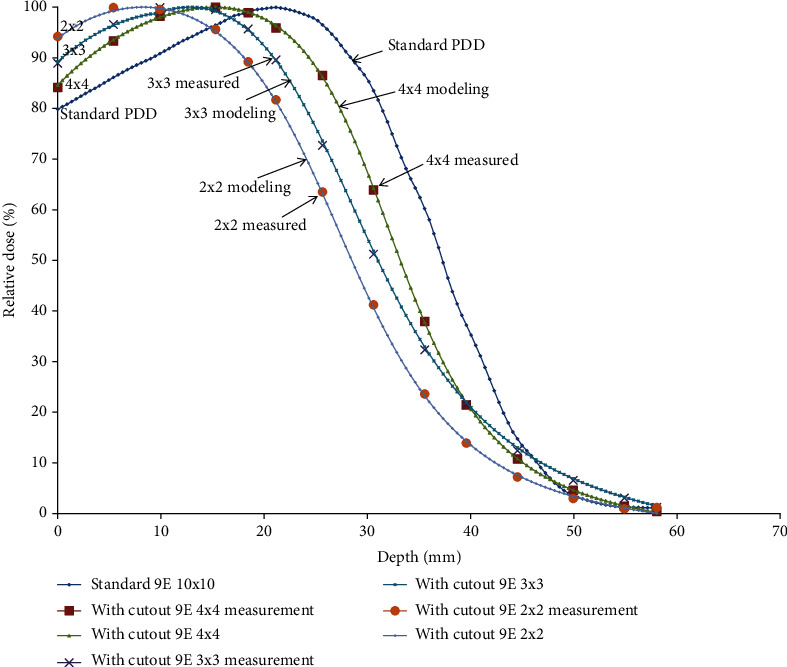
This figure shows the depth dose curves move toward the left significantly when the field size is reduced to less than 4.9 cm in this figure. Thus, the depth dose distribution for small fields is field size-dependent, while for the field that was required for lateral scatter equilibrium (LSE), it is independent of field size.

When the field size is reduced by the electron cutout but keeps a fixed jaw opening, the depth dose curves move toward the left in Figure 5, and the *d*_max_ shifts toward the surface for the smaller fields less than 4 cm^2^.

As the field size is increased in electron beam radiotherapy, the PDD initially increases but becomes constant beyond a certain field size.

Table 4 makes the comparison of S_eq_ and *S*_*eq*_. According to the calculation from S_eq_, the minimum side lengths without changing the standard percent depth dose of 9 MeV and 15 MeV are 4.9 cm and 5.9 cm, respectively.

**Figure 6:**
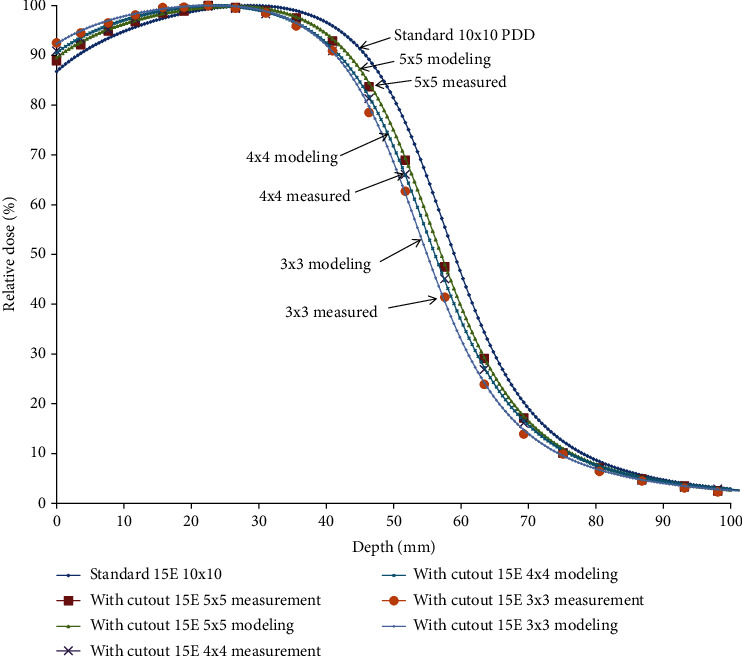
The depth dose curves move toward the left significantly when the field size is reduced to less than 5.9 cm of 15 MeV. It is concluded that for the field that is required for lateral scatter equilibrium (LSE) to remain PDD constant, once the LSE is reached, PDD is independent of field size.

The depth dose curves move toward the left significantly when the field size is reduced to less than 4.9 cm and 5.9 cm of 9 MeV in Figure 5 and 15 MeV in Figure 6, respectively. Thus, the depth dose distribution for small fields is field size-dependent, while for a field that is required for lateral scatter equilibrium (LSE), it is independent of field size.

The essential valuable contribution of this study is the modeling techniques and the establishment of lateral scatter equivalence (LSE) in the clinic using electron beams. The lateral scatter equilibrium for the electron field can be calculated and achieved by using the simple mathematic function presented in this study.

One of the great successes in this study is to provide a home-generated calculation method to determine the equivalent side of a square field that satisfies the establishment of lateral scatter equilibrium so that it keeps the same depth dose distribution for electron cutout fields in clinical use.

The method described in this study provides only for the determination of the equivalent side of a square field but does not include the correction of output factors; therefore, more parameters such as incident fluence, size of the applicator, and electron energy need to be measured to do the correction of output factor [16].

## 5. Conclusions

In this study, we presented a study with an empirical method to model the electron beam percent depth dose (PDD) curve by using the primary function and tail function in radiation therapy. The modeling parameters *N* and *n* can be used to predict the minimal side length when the field size is reduced below that required for lateral scatter equilibrium (LSE) in electron radiation therapy.

Useful criteria have been developed to predict a lack of achievement of lateral scatter equilibrium to check for a field if whether the patient treatment monitor unit needs to be modified or not.

## Figures and Tables

**Table:** (a) This table demonstrates the comparison in fitting the electron energy of 12 MeV with 5 cone sizes from 6cm × 6 cm to 25 cm × 25 cm at SSD = 100 cm

Depth (mm)	6-cone modeling	6-cone standard	Deviation (%)	10-cone modeling	10-cone standard	Deviation (%)	15-cone modeling	15-cone standard	Deviation (%)	20-cone modeling	20-cone standard	Deviation (%)	25-cone modeling	25-cone standard	Deviation (%)
0	82.29	83.83	-1.84	79.93	83.34	-4.09	79.93	82.8	-3.47	79.93	83.24	-3.98	79.93	83.1	-3.81
5	89.59	89.04	0.62	88.49	88.27	0.25	88.49	88.12	0.42	88.49	88.29	0.23	88.49	88.04	0.51
10	94.26	92.65	1.74	93.68	91.81	2.04	93.68	91.8	2.05	93.68	91.75	2.10	93.68	91.65	2.21
15	97.35	95.69	1.73	97.05	95.01	2.15	97.05	94.9	2.27	97.05	94.8	2.37	97.05	94.63	2.56
20	99.3	98.33	0.99	99.17	97.63	1.58	99.17	97.76	1.44	99.17	97.57	1.64	99.17	97.55	1.66
25	100	99.88	0.12	100	99.56	0.44	100	99.71	0.29	100	99.64	0.36	100	99.61	0.39
30	98.92	98.93	-0.01	99.04	99.47	-0.43	99.04	99.11	-0.07	99.04	99.19	-0.15	99.04	99.19	-0.15
35	94.79	93.35	1.54	95.08	94.82	0.27	95.08	93.86	1.30	95.08	94.37	0.75	95.08	93.78	1.39
40	84.52	81.65	3.52	85.13	83.98	1.37	85.13	82	3.82	85.13	82.56	3.11	85.13	81.76	4.12
45	64.59	63.5	1.72	65.55	66.22	-1.01	65.55	63.91	2.57	65.55	64.22	2.07	65.55	62.98	4.08
50	39.93	41.84	-4.57	43.58	44.39	-1.82	40.58	41.83	-2.99	40.58	42.06	-3.52	40.58	40.81	-0.56
55	21.97	22.03	-0.27	21.9	23.63	-7.32	21.39	21.5	-0.51	21.9	21.97	-0.32	21.9	20.99	4.34
60	8.87	8.46	4.85	9.25	9.22	0.33	8.25	8.19	0.73	8.25	8.54	-3.40	8.25	8.09	1.98
65	3.49	3.32	2.71	3.67	3.64	0.82	3.77	3.32	7.53	3.28	3.42	-4.09	3.18	3.27	-2.75
70	2.31	2.16	2.31	2.53	2.31	5.19	2.25	2.23	5.38	2.25	2.22	1.35	2.15	2.14	0.47
75	2.13	2.04	4.41	2.15	2.19	-1.83	2.25	2.15	4.65	2.15	2.14	0.47	2.25	2.06	9.22
80	2.43	2.49	-2.41	2.18	2.14	1.87	2.08	2.13	-2.35	2.28	2.31	-1.30	2.35	2.47	-4.86

**Table:** (b) This table demonstrates the comparison in fitting the five electron energies with cone size 10 cm × 10 cm at SSD = 100 cm

Display (mm)	6 MeV modeling	6 MeV standard	Deviation (%)	9 MeV modeling	9 MeV standard	Deviation (%)	12 MeV modeling	12 MeV standard	Deviation (%)
0	81.45	81.76	-0.38	76.71	79.83	-3.90	79.93	83.34	-4.09
5	96.70	96.35	0.36	85.11	85.66	-0.64	88.65	88.33	0.36
10	99.95	99.71	0.25	92.18	90.93	1.37	93.74	91.84	2.07
15	97.00	95.77	1.29	97.26	96.16	1.15	97.08	95.01	2.18
20	76.57	72.44	5.70	99.89	99.70	0.19	99.17	97.63	1.58
25	33.84	35.63	-5.02	98.10	97.56	0.55	100.00	99.56	0.44
30	9.20	8.74	5.34	86.44	85.75	0.80	99.07	99.53	-0.46
35	2.92	1.30	124.63	58.82	62.81	-6.36	95.08	94.82	0.28
40	1.57	0.84	86.20	29.71	35.45	-16.20	85.13	83.98	1.37
45				14.04	13.41	4.66	65.55	66.22	-1.01
50				5.89	3.44	71.55	40.43	44.03	-8.19
55				2.33	1.25	86.79	21.63	23.15	-6.56
60				1.33	1.08	22.61	11.25	9.22	22.02
65							5.71	3.50	62.96
70									
75									
80									
85									
90									
Display (mm)	15 MeV modeling			15 MeV standard		Deviation (%)	18 MeV modeling	18 MeV standard	Deviation (%)
0	86.37			89.98		-4.02	90.84	87.85	3.41
5	95.05			94.06		1.05	93.33	92.08	1.36
10	97.83			96.45		1.43	95.23	94.29	1.00
15	99.20			97.84		1.39	96.74	95.93	0.85
20	99.85			98.86		1.01	97.95	97.11	0.86
25	99.99			99.66		0.33	98.89	98.34	0.56
30	99.63			99.75		-0.12	99.56	99.20	0.37
35	98.69			99.24		-0.55	99.95	99.74	0.20
40	96.89			97.21		-0.33	99.94	99.98	-0.04
45	93.64			93.30		0.36	99.37	99.16	0.21
50	87.77			86.37		1.62	97.89	96.86	1.06
55	77.30			75.88		1.88	94.76	92.34	2.62
60	60.60			62.10		-2.42	88.57	84.79	4.46
65	40.86			46.10		-11.37	76.99	73.93	4.14
70	25.12			30.31		-17.11	58.70	60.03	-2.22
75	15.51			17.14		-9.53	38.40	44.28	-13.29
80	10.09			8.70		15.93	24.30	29.22	-16.86
85	6.20			4.84		28.09	14.39	16.66	-13.62
90	5.08			3.76		35.11	9.39	8.74	7.38
95	3.87			3.54		9.11	6.49	5.10	27.19
100	3.05			3.47		-12.00	4.71	4.02	17.23
105	2.48			3.38		-26.67	3.56	3.76	-5.29
110	2.07			3.31		-37.45	2.78	3.70	-24.85
115							2.22	3.61	-38.41
120							1.82	3.53	-48.47

**Table 2:** The best fitting of the PDD modeled by the main parameters *N*, *n*, and *μ* in 5 electron energies at a cone size of 10 cm × 10 cm.

Electron energy (MeV)	*N*	*n*	*μ*
6	3.1	90	0.001
9	6.2	210	0.0007
12	8.9	290	0.0005
15	12.3	400	0.0003
18	15.1	470	0.0002

Note: *N* is a scalar of harden factor, *n* is a scalar of the spread factor while *μ* is the linear attenuation factor in the unit of mm^−1^. The *x* is a real number on the horizontal axis in the unit of mm, *x* also denotes the depth in water.

**Table 3:** The maximum ionizing depth, dose maximum depth, and mean energy in five different electron energies with 10 cm × 10 cm, *E*_0_ (*E*_0_ = 2.33 MeV/cm · *R*_50_) is shown. The depth at which the dose is 50% of the maximum dose is defined as *R*_50_ in this study.

Nominal energy (MeV)	*E*_0_ (MeV)	*R*_max_/*d*_max_ (mm)	*R*_90_ (mm)	*R*_50_ (mm)	*R*_p_ (mm)	X-ray contamination (%)
6	5.41	11.1/12.1	16.9	23.2	30.2	0.7
9	8.66	20.6/21.5	28.3	37.1	45.1	1.2
12	11.27	26.1/27.1	37.4	48.3	58.7	2.1
15	14.04	27.1/28.4	46.1	60.3	73.7	2.6
18	17.03	24.3/25.2	56.8	73.1	88.7	3.5

**Table 4:** The comparison ofS_eq_and*S*_*eq*_, the minimum side lengths to remain the standard percent depth doses of 9 MeV and 15 MeV unchanged are 4.9 cm and 5.9 cm, respectively.

Energy (MeV) (nominal)	*E*_0_ (MeV)	*n*	*N*	Slope (80%-20%)	tan^−1^ (slope)	*μ*	ln(*μ*)	S_eq_ (mm, this study)	$S_{eq}=1.58\sqrt{E_{0}}$ (mm)
6	5.41	90	3.1	-8.6593	-1.45582	0.001	-6.9077	38.13	36.75
9	8.66	210	6.2	-5.7176	-1.39765	0.0007	-7.2644	49.17	46.50
12	11.27	290	8.9	-4.5559	-1.35473	0.0005	-7.6009	53.98	53.04
15	14.04	400	12.3	-3.3749	-1.28273	0.0003	-8.1117	59.33	59.20
18	17.03	470	15.1	-2.6474	-1.20964	0.0002	-8.5171	62.10	65.20

## Data Availability

The data used to support the findings of this study are available from the corresponding authors upon request.
